# Overexpression of LCMR1 is significantly associated with clinical stage in human NSCLC

**DOI:** 10.1186/1756-9966-30-18

**Published:** 2011-02-09

**Authors:** Liangan Chen, Zhixin Liang, Qing Tian, Chunsun Li, Xiuqing Ma, Yu Zhang, Zhen Yang, Ping Wang, Yanqin Li

**Affiliations:** 1 Department of Respiratory Diseases, Chinese PLA General Hospital, Beijing 100853, PR China

## Abstract

**Background:**

Lung cancer is one of the most common human cancers and the leading cause of cancer death worldwide. The identification of lung cancer associated genes is essential for lung cancer diagnosis and treatment.

**Methods:**

Differential Display-PCR technique was used to achieve the novel cDNA, which were then verified by real-time PCR. Northern blot was utilized to observe the expression of LCMR1 in different human tissues. 84 cases human NSCLC tissues and normal counterparts were analyzed for the expression of LCMR1 by immunohistochemistry.

**Results:**

A novel 778-bp cDNA fragment from human large cell lung carcinoma cell lines 95C and 95D was obtained, and named *LCMR1 *(Lung Cancer Metastasis Related protein 1). LCMR1 was differentially expressed in different human tissues. LCMR1 was strongly overexpressed in NSCLC and its expression was significantly associated with clinical stage.

**Conclusion:**

Our data indicated that *LCMR1*, strongly overexpressed in NSCLC, might have applications in the clinical diagnosis and treatment of lung cancer.

## Introduction

The development of new therapeutics and diagnostics of cancer rely on the understanding of carcinogenesis mechanisms. Genes dysregulated significantly in tumor tissues compared with their normal counterparts are always considered as biomarkers or closely associated with carcinogenesis. Over the past two decades plentiful efforts have been devoted to the identification of genes involved in cancer development [1].

Many approaches have been used to compare gene expression between two different physiological states. Differential Display (DD) is a useful method to compare patterns of gene expression in RNA samples of different types or under different biological conditions [2,3]. The technique produces partial cDNA fragments by a combination of reverse transcription and PCR of randomly primed RNA. Changes in the expression level of genes are identified after separation of the cDNA fragments produced in an arbitrarily primed polymerase chain reaction on a sequencing-type gel. Combined with RNA expression verification, Differential Display is a powerful method for generating high confidence hits in the screening of hundreds of potential differentially expressed transcripts.

Lung cancer is one of the most common human cancers and the leading cause of cancer death worldwide [4,5]. With the same genetic backgrounds but different metastatic potential, 95C and 95D cell lines were subcloned from a poorly differentiated human large cell lung carcinoma cell line PLA-801 by Dr. Lezhen Chen (Department of Pathology, Chinese PLA General Hospital), which were suitable for Differential Display analysis. Nude mice incubated with 95D cells showed earlier and more metastasis than incubated with 95C cells [6,7]. Although the importance of tumorigenesis has been realized and studied, limited knowledge is known about its associated genes and signal networks. Understanding further more players and intrinsic processes involved in carcinogenesis could lead to effective, targeted strategies to prevent and treat cancer.

In the present study, we found that *LCMR1 *was expressed significantly higher in 95D cell line compared to 95C using a combination of DD-PCR and real-time PCR. We then investigated its expression in various human tissues by northern blot. Recombinant LCMR1 protein was expressed and its specific polyclonal antibody was generated. To examine its involvement in carcinogenesis, 84 specimens of NSCLC patients were examined for the expression of LCMR1 by immunohistochemistry analysis. Our results strongly suggested that LCMR1 was significantly overexpressed in human NSCLC and its expression was closely associated with clinical stage of patients with NSCLC, which may have applications in lung cancer diagnosis and treatment.

## Materials and methods

### Cell lines

95C and 95D cell lines were subcloned from a poorly differentiated human large cell lung carcinoma cell line PLA-801 and kindly provided by Dr. Lezhen Chen (Department of Pathology, Chinese PLA General Hospital, China). Both cell lines were cultured in RPMI 1640 medium, supplemented with 10% fetal bovine serum, 100 μg/ml penicillin, and 100 μg/ml streptomycin at 37°C in a humidified 5% CO_2 _incubator.

### RNA extraction and cDNA synthesis

Total RNA was prepared using Trizol reagent (Invitrogen, CA, USA) according to the manufacturer's instructions. RNA was treated with RNase (Invitrogen) in the presence of 50 μM T7 (dT12) AP1, T7 (dT12) AP5 and T7 (dT12) AP8 primers in 20 μl RT buffer (1× Superscript II RT buffer, 10 mM DTT, 0.025 mM dNTP), at 25°C for 5 minutes, followed by 50°C for 50 minutes. Reverse transcriptase was inactivated at 70°C for 15 minutes.

### Differential display and full-length gene cloning

Differential display was performed using Hieroglyph mRNA Profile Kit (Beckman, CA, USA). Briefly, PCR amplification was done using 1.5 μl of the cDNA, primed with arbitrary P primer and anchored T primer. Amplification at (95°C 2 minutes) 1 cycle, (92°C for 15 seconds, 50°C for 30 seconds, 72°C for 2 minutes) 4 cycles, (92°C for 15 seconds, 60°C for 30 seconds, 72°C for 2 minutes) 30 cycles, followed by a final extension at 72°C for 7 minutes on a GeneAmp PCR system 9600 (Perkin-Elmer, Norwalk, USA). Following amplification of randomly primed mRNAs by RT-PCR, the cDNA products were heated at 95°C for 2 minutes and separated on a denaturing 5.6% polyacrylamide gel at 55°C for 5 hours using a Genomyx LR DNA Sequencer (Beckman), under 3000 V. Bands exclusively present in either of two samples were considered as candidates of differentially expressed transcripts, which were excised, eluted, re-amplified, and subcloned into the T easy vector (Promega, San Luis Obispo, CA, USA). The sequence reactions were performed by Invitrogen. Sequence homology to published database was analyzed with the BLAST program at the internet site of NCBI (National Center for Biotechnology Information). 5'-RACE (rapid amplification of cDNA 5' ends) and 3'-RACE were used to isolate the complete cDNA. The human Marathon-ready cDNA (Clontech, Heidelberg, Germany) served as the template.

### Real-time quantitative reverse transcription polymerase chain reaction

We measured LCMR1 gene expression in 95C and 95D cell lines by real-time quantitative RT-PCR in an ABI PRISM 7500 Sequence Detection System. The real-time RT-PCR allows, by means of fluorescence emission, the identification of the cycling point when PCR product is detectable. The Ct value inversely correlates with the starting quantity of target mRNA. Measurements were performed in duplicate and the controls were included in which the reaction mixture contained no cDNA. The amount of target mRNA after normalized to the loading control β-actin was calculated by the Ct method. Primers for β-actin and LCMR1 mRNAs were chosen using the Primer Express 2.0 software (Applied Biosystems, Foster City, USA). Primers for LCMR1 were: 5'-AACAGAGCCGTACCCAGG AT-3' (Forward) and 5'-GGGTGGTCTGGACATTGTC -3' (Reverse). Primers for β-actin were: 5'-CATGTACGTTGCTATCCAGGC-3' (Forward) and 5'-CTCCTTAATGTCACGCAC GAT- 3' (Reverse). Primers were synthesized by Invitrogen.

### RNA expression analysis by northern blot in human normal tissues

LCMR1 expression was analyzed by multiple tissue northern blots (MTN) in a panel of following normal tissues (Clontech): brain, heart, skeletal muscle, colon, thymus, spleen, kidney, liver, small intestine, placenta, lung, and peripheral blood leukocytes. Hybridization was performed using 25 ng of a gene-specific 32P-labeled DNA probe derived from LCMR1 cDNA. This gene-specific cDNA fragment was radiolabelled using a Prime-A-Gene Labeling System (Promega), hybridized overnight at 68°C using ExpressHyb Hybridization Solution (Clontech), washed, and exposed to Kodak XAR-5 X-ray film with an intensifying screen (Eastman Kodak Co, Rochester, NY, US).

### Expression and polyclonal antibodies preparation of LCMR1 protein

The plasmid pGEX-5T-LCMR1 was constructed. The GST-LCMR1 protein expression was induced by adding 0.6 mM IPTG to the transformed E. coli and the bacteria were incubated at 20°C for 4 hours. The degree of expression was evaluated by sodium dodecyl sulfate-polyacrylamide gel electrophoresis (SDS-PAGE). The GST-LCMR1 fusion protein was purified by affinity chromatography using glutathione-agarose resin (GE Healthcare). The New Zealand white rabbits were given intradermal injections of purified GST-LCMR1 fusion protein and the antibody against LCMR1 was prepared. The titer of antiserum was determined by an indirect ELISA.

### Cases and Clinical Data

We studied a consecutive series of 84 cases primary NSCLC cancers diagnosed and treated between 2005 and 2007 at the Department of thoracic surgery, Chinese PLA General Hospital, Beijing, China. None of the patients had received radiotherapy or neoadjuvant therapy before surgery. Metastatic lymph nodes of 51 cases in this group were also examined for the expression of LCMR1. The duration of 65 cases follow-up ranged from 5 to 39 months (median, 31 months). Tumor characteristics, including histologic grade, lymph node status, and clinical stage, were routinely assessed by pathologists.

### Immunohistochemical analysis

The sections were dewaxed with xylene and rehydrated through an ethanol gradient into water. After endogenous peroxidase activity was quenched with 3% H_2_O_2 _for 30 minutes, sections were digested with 0.1% trypsin at 37°C for 20 minutes. After phosphate-buffered saline (PBS) washing, nonspecific antibody binding was blocked by incubating the slides with 10% normal goat nonimmune serum for 30 minutes at 37°C. Sections were incubated at 4°C overnight with the self-made rabbit polyclonal primary antibody against human LCMR1 at a 1:200 dilution. After PBS washing, sections were incubated with biotinylated secondary antibody for 30 minutes at 37°C and then with horseradish peroxidase-labeled streptavidin for 30 minutes at 37°C. After PBS washing, sections were developed using 3,3V-diaminobenzidine (Sigma-Aldrich). Sections were washed in running tap water and lightly counterstained with hematoxylin, followed by dehydration and coverslip mounting. Negative controls were obtained by omitting the primary antibody [8].

### Statistical analysis

The criterion for a positive reaction was a single epithelial cell with yellow particles in its plasma membrane and cytoplasm. Immunostaining was assessed in a blinded manner for extent and intensity. In brief, a sample with no positive epithelial cells was scored as 0, that with less than 25% total positive epithelial cells was scored as 1+, that with positive epithelial cells accounting for more than 25% but less than 50% of the total was scored as 2+, that with more than 50% but less than 75% positive cells was scored as 3+, and that with more than 75% positive cells was scored as 4+. The intensity of immunostaining was scored semiquantitatively as follows: no obvious yellow particle in epithelial cell plasma membrane or cytoplasm as 0; with light yellow particles as 1+ (weak); with general yellow particles as 2+ (moderate); and with deep yellow particles as 3+ (strong). For each case, an immunoscore was calculated as the product of 2 scores assessed separately. Statistical analysis was performed using SPSS 17 software (SPSS, Inc, Chicago, IL, USA). The differential expression of LCMR1 protein between tumorous tissues and normal tissues was determined by Mann-Whitney U-test. The correlations between LCMR1 expression and clinicopathologic characteristics were analyzed using Pearson χ^2 ^analysis. The influence of each variable on the expression of LCMR1 was assessed by logistic regression analysis. In survival analysis, Kaplan-Meier curves were drawn, univariate and multivariate analyses in a Cox proportional hazards model were used for LCMR1 scores. All statistical tests were 2-sided, and P values of 0.05 or less were considered statistically significant.

## Results

### Cloning and identification of a novel gene differentially expressed in 95C and 95D cell lines using DD-PCR

**Figure 1:**
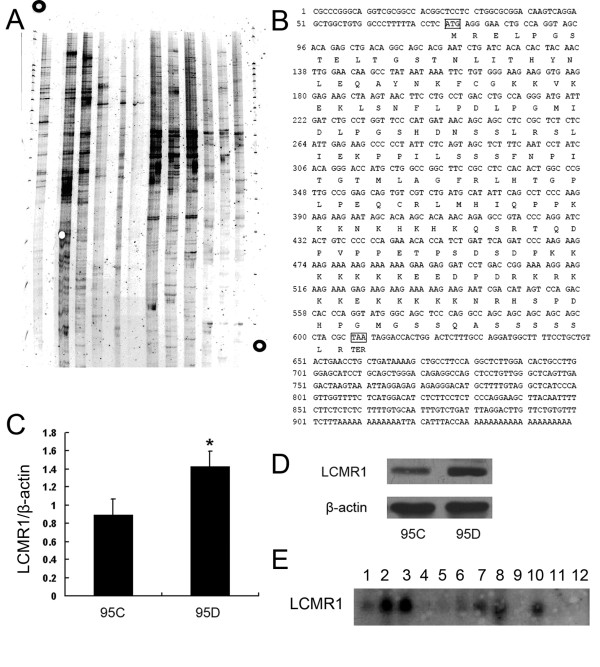
**Cloning of a novel gene, *LCMR1***. (A) Electrophoresis result of DDRT-PCR in 95C and 95D cells. (B) Nucleotide and amino acid sequences of LCMR1 cDNA. *LCMR1 *contains a 74-bp 5'- UTR, a 949-bp ORF, and a 341-bp 3'-UTR. Inframe termination (TER) codons are located at nt positions 606-608. LCMR1 encodes a 177 aa protein. (C) LCMR1 mRNA expressions in 95C and 95D cells were examined by real-time quantitative RT-PCR. LCMR1 gene expression level in 95D cells was significantly higher than in 95C cells. (*, *P *< 0.01) (D) LCMR1 protein expression in 95D cells was significantly higher than in 95 C cells, examined by western blot. (E) LCMR1 was differentially expressed in the various human tissue distributions by multiple tissue northern blot (MTN). Numbers indicate tissue types in columns. 1: Brain, 2: Heart, 3: Skeletal muscle, 4: Colon, 5: Thymus, 6: Spleen, 7: Kidney, 8: Liver, 9: Small intestine, 10: Placental, 11: Lung, 12: Leukocyte.

In order to find lung cancer metastasis related genes, the DD-PCR method was used to identify genes differentially expressed in human 95C and 95D cell lines, which have the same genetic backgrounds but different metastatic potential. Several cDNAs were found expressed differentially in these two cells (Figure 1A). These fragments were subcloned into T easy vector, sequenced, and analyzed for nucleotide and amino acid homology in the GenBank database. Of these, a 778 bp cDNA fragment, designated as P9, expressed higher in 95D cells than in 95C cells, did not show a significant homology with any nucleotide/amino acid sequence in the database, but has many supports of EST. After alignment in Genbank Genomic Database, we found this fragment existed in chromosome 11 discontinuously. These suggested that this cDNA might code a novel gene, and thus was selected for further studies. RACE (rapid amplification of cDNA ends) was used to get the complete cDNA. Using P9 as a probe, we obtained the full-length 949 bp cDNA, nominated as *LCMR1 *(Lung Cancer Metastasis Related gene 1) (Figure 1B). We submitted this result in 2002 and acquired the Genbank accession number as AY148462.

*LCMR1 *cDNA was found to be a novel sequence without any homology with any known nucleotide/amino acid sequence in the database. *LCMR1 *cDNA was found to be located on human 11q12.1 chromosome locus. Analysis of *LCMR1 *cDNA using the DNA analysis program revealed that it has an ORF starting with an ATG initiation codon at nucleotide 75-77 with a termination codon at nucleotide 606-608. It has a 5'-UTR of 74 bp and a 3'-UTR of 341 bp. Analysis of the predicted peptide using Vector NTI DNA analysis software program revealed that the predicted peptide of *LCMR1 *has 177 amino acid residues with a calculated molecular mass of 19,950 Da and an isoelectric point of 10.01.

### Confirmation of LCMR1 differentially expressed in 95C and 95D cell lines by real-time PCR and western blot

In order to further confirm the difference of *LCMR1 *gene expression between 95C and 95D cell lines, we compared *LCMR1 *mRNA expression in these two cell lines by real-time quantitative RT-PCR. As shown in Figure 1C, L*CMR1 *gene expression level in 95D cells was significantly higher than in 95C cells. Western blot analysis with LCMR1 antibody generated as followed procedure also showed the consistent result (Figure 1D).

### Expression of LCMR1 in Various Human Tissues by Northern blot

Multiple tissue northern blot (MTN) was adopted to determine the various tissue distribution of human LCMR1 in RNA level. As shown in Figure 1E, LCMR1 was differentially expressed in all the tissues investigated, with high expression detected in the heart, skeletal muscle, kidney, liver, and placental tissue, while low or hardly detected in others.

### Expression and polyclonal antibodies preparation of recombinant LCMR1 protein

**Figure 2:**
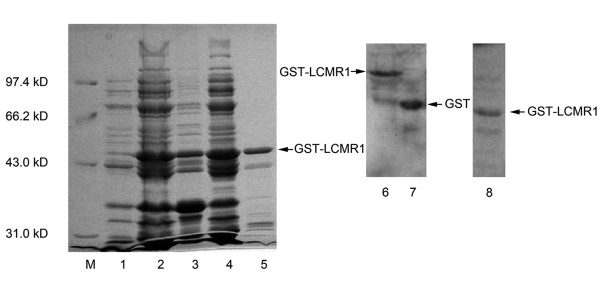
**Recombinant LCMR1 protein expression and polyclonal antibody preparation**. M, protein marker; lane 1, pGEX-5T-LCMR1 before induction in E.coli; lane 2, pGEX-5T-LCMR1 after induction in E.coli; lane 3, precipitation after E.coli lysis; lane 4, clear supernatant after E.coli lysis; lane 5, GST-LCMR1 after purification; lane 6, GST-LCMR1 fusion protein recognized by GST antibody; lane 7, GST protein recognized by GST antibody; lane 8, GST-LCMR1 fusion protein recognized by LCMR1 polyclonal antibody. (lane 1-5, SDS-PAGE; lane 6-8, western blot)

The full length of human LCMR1 CDS region was cloned into pGEX-5T. Under optimized induction condition, GST-LCMR1 fusion protein was highly expressed after induction at 20°C with 0.6 mM IPTG for 4 hours in *E.coli*. With purification using glutathione-agarose resin, the fusion protein was separated from those unwanted proteins (Figure 2, lane 5). The GST-LCMR1 fusion protein and GST was recognized clearly by specific GST antibody (Figure 2, lane 6 and 7). Then the purified fusion protein was excised and used to immunize New Zealand rabbits. ELISA was used to determine the titers of the obtained antibody and the antibody at different dilutions (1000 to 100,000) was reacted with an equal amount of the recombinant protein (data not shown). The antibody specificity was examined by western blot (Figure 2, lane 8).

### Overexpression of LCMR1 protein in human NSCLC by immunohistochemistry analysis

**Figure 3:**
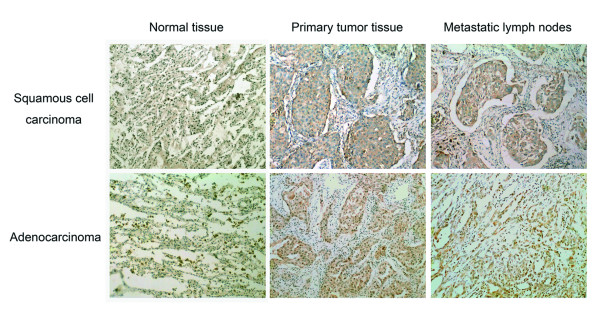
**LCMR1 expression in human NSCLC**. Compared with adjacent normal tissues, LCMR1 was significantly overexpressed in primary tissues and metastatic lymph nodes of patients with NSCLC respectively by immunohistochemistry analysis. (Magnification: ×100)

There existed various degrees of background staining that may be caused by tissue processing, such as fixation and embedding. Because such background staining is almost nonspecific, occurring in the stromal tissue (including lymphocytes), we avoided it by counting only positive epithelial cells. Also, the edge effect was regarded as negative. Immunohistochemistry analysis results showed that the expression of LCMR1 was significantly higher in primary tumor tissues (84 cases) and metastatic lymph nodes (51 cases) of NSCLC patients, compared with its weak expression in adjacent benign tissues respectively (*P *< 0.001) (Figure 3, Table 1). There is no difference in the expression of LCMR1 between primary tumor tissues and metastatic lymph nodes (data not shown). Moreover, immunostaining showed LCMR1 was expressed mostly in the cytoplasm of cells.

**Table 1:** Expression of LCMR1 in primary tumor tissues, adjacent normal tissues and metastatic lymph nodes.

Expression of LCMR1 between two groups	*P*
primary tumor tissues *vs *paired adjacent normal tissues (84 cases)	0.000
metastatic lymph nodes *vs *paired normal tissues (51 cases)	0.000
primary tumor tissues *vs *paired metastatic lymph nodes (51 cases)	0.678

### Association between LCMR1 expression and clinical stage and prognosis of human NSCLC

Patient characteristics, including gender, age (range, 32-77 years; median, 59 years), smoking status, pathological type, histologic grade, lymph node metastasis, and clinical stage (classified according to the 2003 TNM classification of the International Union Against Cancer) are recorded in Table 2. Statistical analysis results showed that LCMR1 expression was significantly associated with clinical stage of these NSCLC patients (*P *< 0.05), but no significant association was found between LCMR1 expression and other clinicopathologic parameters such as gender, age, smoking status, pathological type, and histologic grade (Table 2). We further used the stepwise forward logistic regression analysis to assess the effects of clinical stages on LCMR1 expression. Logistic regression analysis revealed that an increased clinical stage was significantly associated with high LCMR1 expression (OR = 3.410, *P *= 0.026) (Table 3). The expression of LCMR1 protein in metastatic lymph nodes had no relationship with the clinic features of NSCLC patients (data not shown).

**Table 2:** Correlations between LCMR1 expression and clinicopathologic characteristics of human NSCLC.

	n	LCMR1 expression		*P*
			
		Negative	Positive	
Gender				
Male	61	12	49	0.147
Female	23	8	15	
Age(y)				
≥65	22	4	18	0.471
<65	62	16	46	
Smoking status				
Yes	45	10	35	0.714
No	39	10	29	
Pathological type				
Adenocarcinoma	41	10	31	0.614
Squamous cell carcinoma	40	10	30	
Adenosquamous carcinoma	3	0	3	
Histologic grade				
PD	28	6	22	0.918
MD	45	11	34	
WD	11	3	8	
Lymph node metastasis				
Yes	62	12	50	0.108
No	22	8	14	
Clinical stage				
I-II	40	14	26	0.022
III-IV	44	6	38	

Abbreviations: WD, well differentiated; MD, moderately differentiated; PD, poorly differentiated.

**Table 3:** Logistic regression analysis.

	Wald χ^2^	*P*	OR
TNM stage	6.995	0.026	3.410

### Survival analysis

**Figure 4:**
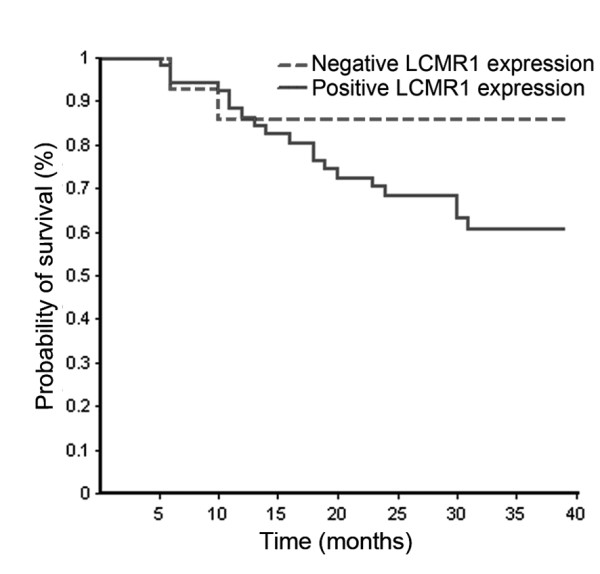
**Kaplan-Meier analysis of 65 cases follow-up**. The survival curve showed increased difference in survival rates between patients with high-level LCMR1 protein expression and patients with low-level LCMR1 expression, with overall survival time extension.

Kaplan-Meier analysis of 65 cases of this group, with a median follow-up of 31 months, showed increased difference in survival rates between patients with high-level LCMR1 protein expression and patients with low-level LCMR1 expression, with overall survival time extension (Figure 4). But no statistical significance was observed in overall survival (OS) and progression-free survival (PFS) of these NSCLC patients using univariate survival analysis and multivariate survival analysis and COX proportional hazard model analysis (data not shown).

## Discussion

Tumor development is a complex and multistage process involving many genetic alterations. It is essential to explore the molecular mechanisms of tumor formation and progression to develop rational approaches to the diagnosis and therapy of cancer, therefore, identifying dysregulated genes and proteins in neoplasms are critical. 95C and 95D cells, subcloned from poorly differentiated human large cell lung carcinoma cell line PLA-801, were of different metastatic potential, while they came from the same patient and had similar genetic background [6,7]. We performed DD-PCR between these two cell lines to find some novel genes involved in lung cancer, and obtained several cDNA fragments expressed differentially between 95C and 95D cells. All these cDNA fragments were subcloned, sequenced, searched for homology with known genes in the database. Among these, the P9 cDNA fragment did not reveal homology with any known gene in the database. Screening the human cDNA library with this specific cDNA fragment yielded a full-length *LCMR1 *cDNA, comprised of 949 nucleotides, having an ORF encoding for a 177 amino acids peptide. Both nucleotide and amino acid sequences did not show homology with any gene reported previously in the database, indicating it to be a novel cDNA. It has a 5'-UTR of 74 bp and a 3'-UTR of 341 bp. The UTRs may be involved in stabilizing mRNA for translation regulation. Most eukaryotic mRNAs possess short 5'-UTRs of 20-100 nucleotides that enable efficient cap-dependent ribosome scanning [9]. We submitted this result in 2002 and acquired the Genbank accession number as AY148462. We further confirmed the different expression of *LCMR1 *between 95C and 95D cell lines by real-time quantitative RT-PCR and western blot analysis. To understand the function of LCMR1, we first investigated LCMR1 mRNA expression in different human normal tissues by northern blot analysis. The results showed that LCMR1 was detected in various kinds of human tissues with different expression levels, which suggested the functions of LCMR1 might vary in different tissues.

To understand the function of *LCMR1*, we investigated LCMR1 protein expression in 84 cases human NSCLC tissues by immunohistochemistry analysis. The results showed that LCMR1 was strongly overexpressed in NSCLC tissues and metastatic lymph nodes, compared with adjacent normal tissues. To find out the correlations between LCMR1 expression and the biologic behavior of NSCLC, we studied clinical data, including gender, age, smoking status, pathological type, histologic grade, lymph node metastasis, and clinical stage. Analysis of gender, age, smoking status, pathological type, histologic grade, and lymph node metastasis revealed that none of them showed a significant correlation with high LCMR1 protein expression. However, high LCMR1 expression was closely associated with clinical stage (*P *= 0.022). Logistic regression analysis result also showed that clinical stage was significantly associated with LCMR1 expression (OR = 3.410, *P *= 0.026). These results suggested the critical role of LCMR1 in human NSCLC development. The Kaplan-Meier analysis of 65 cases of this group showed that LCMR1 expression had no significance with overall survival, which may be due to short follow up periods. However, it showed the tendency that positive LCMR1 expression was associated with poor survival. The results showed that there is no difference between the levels of LCMR1 expression in the primary tumors with or without metastasis, neither between metastatic sites and primary sites. The study on more pathological specimens would shed light on this relationship.

LCMR1 was also found to be a member of mammalian Mediator subunits, called MED19 [10,11]. The mediator complex is a large collection of DNA binding transcriptional activators through the action of an intermediary multiprotein coactivator, which controls the transcription of eukaryotic protein-coding genes with RNA polymerase II (pol II) [12]. Specific mediator subunits are dedicated to regulate distinct expression programs via interactions with relevant gene-specific transcriptional activators, which lead to activation of transcription at the target gene. It has been reported that normal function of activators, such as VP16 and p53, interact with different Mediator subunits [13]. Recently, it was reported that MED19 (LCMR1) and MED26 subunits as direct functional targets of the RE1 Silencing Transcription Factor, REST, facilitated REST-imposed epigenetic restrictions on neuronal gene expression [14]. Mediator serves as a key cofactor and integrator of signaling in many transcriptional activations and pathways. Exact temporal and spatial regulation of the transcription of genes is vital to the execution of complex gene functions in response to growth, apoptosis, developmental and homeostatic signals, etc [15,16]. MED1 has been found to play an important coregulatory role in the development and progression of lung adenocarcinoma [17]. Although Mediator complex has been studied for many years, limited knowledge was known about MED19/LCMR1. Our results suggested that LCMR1 has an important clinicopathological role in the lung cancer. It will be of considerable interest to further understand these interactions and elucidate the intrinsic mechanisms, since one of the most important reasons of cancer development is the dysfunction of transcriptional regulation associated genes.

In conclusion, we are the first to identify *LCMR1 *gene. The present study revealed that the expression of LCMR1 was significantly up-regulated in primary tissues and metastatic lymph nodes of patients with NSCLC, compared with adjacent normal tissues. Its role in carcinogenesis needs to be further investigated. The strong correlation between LCMR1 expression and clinical stage indicates that LCMR1 could serve as a biomarker for judging the level of malignancy of lung cancer, which may guide the development of anticancer therapy.

## Abbreviations

CDS: coding Sequence; DD: differential display; ELISA: enzyme-linked immunosorbent assay; ETS: expressed sequence tag; LCMR1: lung cancer metastasis related protein 1; NSCLC: non-small cell lung cancer; OS: overall survival; PBS: phosphate-buffered saline; PFS: progression-free survival; RT-PCR: reverse transcriptase-polymerase chain reaction; UTR: untranslated Regions.

## Competing interests

The authors declare that they have no competing interests.

## Authors' contributions

LC and ZL are joint first-authors, and contributed equally to this study. LC conceived of the work. LC and QT carried out the gene cloning and RNA expression analysis of LCMR1 in normal human tissues. ZL prepared GST-LCMR1 protein and antibody. CL participated in the qPCR and drafted the manuscript. ZL and XM performed immunohistochemistry analysis. CL and YL carried out qPCR. YZ, ZY, and PW collected the cases and sections. LC participated in the design and coordination and supervised the whole study. All authors read and approved the final manuscript. All authors read and approved the final manuscript.
